# Advancing the status of nursing: reconstructing professional nursing identity through patient safety work

**DOI:** 10.1186/s12913-019-4222-y

**Published:** 2019-06-24

**Authors:** Frode Heldal, Trond Kongsvik, Erna Håland

**Affiliations:** 1 NTNU Business School, Norwegian University of Science and Technology (NTNU), NO-7491 Trondheim, Norway; 2 Department of Industrial Economics, Norwegian University of Science and Technology (NTNU), NO-7491 Trondheim, Norway; 3 Department of Education and Lifelong Learning, NO-7491 Trondheim, Norway

**Keywords:** Patient safety, Audit systems, Professional nursing identity, Care

## Abstract

**Background:**

Recent decades have seen increased attention to patient safety in health care. This is often in the form of programmes aiming to change professional behaviours. Health professionals in hospitals have traditionally resented such initiatives because patient safety programmes often take a managerialist form that may be interpreted as a challenge to professional identity. Research, however, has mostly paid attention to the role of physicians. This study aims to highlight how such programmes may affect professional nursing identity.

**Methods:**

We qualitatively investigated the implementation of a patient safety programme in Norway, paying attention to changes in nurses’ practices and values. Based on purposive sampling, two group interviews, four individual interviews and five hours of observational studies were conducted in a hospital department, involving ten nurses and three informants from the hospital management. Interviews were conducted in offices at the hospital, and observations were performed in situ. All the interviews lasted from one to one and a half hours, and were recorded and transcribed ad verbatim. Data was analysed according to ad-hoc meaning generation.

**Results:**

The following analytical categories were developed: reconstructing trust, reconstructing work, reconstructing values and reconstructing professional status. The patient safety programme involved a shift in patient safety-related decisions, from being based on professional judgement to being more system based. Some of the patient safety work that previously had been invisible and tacit became more visible. The patient safety programme involved activities that were more in accordance with the ‘cure’ discourse than traditional ‘care’ work within nursing. As a result, this implied a heightened perceived professional status among the nurses. The safety programme was – contrary to the ‘normal’ resistance against audit systems – well received because of the raised perceived professional status among the nurses.

**Conclusions:**

Reconstructing trust, work, values and status, and even the profession itself, is being reconstructed through the work involved in implementing the procedures from the safety programme. Professional knowledge and identity are being challenged and changed, and what counts as good, professional nursing of high quality is being reconstructed.

**Electronic supplementary material:**

The online version of this article (10.1186/s12913-019-4222-y) contains supplementary material, which is available to authorized users.

## Background

The starting point for this article is that professional identity is not fixed but context dependent [1, 2]. Changes in the framework conditions of health professionals (e.g. changed emphasis on patient safety) might therefore affect professional priorities, work practices and what is considered to be valid knowledge, and thus professional identity. We see professional identity as “an awareness of the role and functions that one performs or is expected to perform in a social context as a member of a particular profession” [3].

Contextual changes of relevance to nurses’ professional identity include the application of management ideas from the private sector to public health systems, such as lean production [4] and total quality management [5], as well as the broad philosophy of new public management [6] involving the principles of accountability and the use of indicators and other metrics for the monitoring and measurement of quality. Although measurement of the quality of care has been regarded as challenging, prevalence measurement schemes exist for problems such as pressure ulcers, malnutrition and falls [7], areas that involve nurses’ patient safety work. In the project forming the empirical basis of this paper, we studied the implementation of a national patient safety programme in Norway called ‘In Safe Hands 24-7’ in a department in a regional hospital and how it affected nursing identity. The programme involved new ways of monitoring and measuring patient safety.

### Patient safety

Patient safety is about not harming patients. There are many formal definitions of patient safety, and the following covers many aspects of the different conceptualisations: “the reduction of risk of unnecessary harm associated with healthcare to an acceptable minimum” [8]. Increased attention to patient safety in health care has been apparent in recent decades. This is partly related to more structured efforts to measure patient harm, which have revealed the magnitude of the problem. The insights from the report ‘To err is human’, and similar reports from other countries, instigated a variety of programmes, on both the global and national levels [9]. Review studies show that the median incidence rate of in-hospital adverse events ranges from 9 to 10%, with considerable variations between the reviewed studies [10, 11]. In Norway, 13.7% of hospital stays in 2017 involved an adverse event, measured by the Global Trigger Tool methodology [12, 13].

Patient safety has traditionally been implicit in the nursing profession, integrated into day-to-day nursing practices. It has been part of the 24/7 care for patients, in which patients’ general well-being is at the centre of attention [14]. This implicit attention to patient safety has also been evident in education and nursing curriculum guidelines [15, 16]. Nursing educators have advocated patient safety as a basic and integrated foundation of nursing and claimed that a ‘compartmentalisation’ of the issue is not recommended [15], for example, by implementing programmes detached from local conditions. This is in line with the argument that nurses should treat patients holistically, based on their knowledge of individual patient care needs [17], often contrasted with the physicians’ ‘cure’ discourse, where curing the disease is central.

### Study context and aim

The World Health Organization (WHO) has run a patient safety programme since 2004 and has launched several policies and campaigns [18, 19]. Based on the WHO programme, the Ministry of Health and Care Services in Norway initiated a national patient safety campaign in 2011, which evolved into a five-year programme from 2014 called ‘In Safe Hands 24-7’. From 2019, a department in the Norwegian Directorate of Health will run the initiative. The initiative aims to reduce patient harm, build sustainable structures for patient safety and improve the patient safety culture [20]. The initiative describes 16 priorities, including safe surgery, medical reconciliation, the prevention of falls in healthcare institutions, prevention of malnutrition, and prevention of pressure ulcers. The priorities were chosen based on their potential to improve patient safety, the available and documented measures and the availability of methods for evaluating effect. The initiative provides a range of tools and improvement methods for health care providers, in both hospitals and primary care, and regional and local programme managers support and guide the activities. There has been considerable activity at the hospital level, and several national learning networks have been established for the priorities. An evaluation of the initiative showed that respondents from specialised care regard the priorities as important, but that the number of priorities is considered demanding [21].

The department included in this study began implementation of the national patient safety initiative in 2012 when a new position as patient safety nurse was established. The nurse’s responsibilities have evolved gradually, and at the time of our study included the preparation of incident statistics at the ward level in areas defined by the patient safety programme. The patient safety nurse presented the incident numbers at monthly ‘huddleboard meetings’ at which incident developments within the ward were compared with those of other wards in the department. The huddleboards were used to address the status of the defined risk areas and action plans for improvement. Special attention was placed on falls, pressure ulcers, and malnutrition. A quality improvement strategy was the basis for the activities, and the ‘Plan-Do-Check-Act’ circle was actively used in communication with the wards. Information on the initiative was given in general department meetings, and head nurses were responsible for following up on the initiative at the ward level, both in meetings and in the day-to-day activities. The initiative involved new tasks for nurses, related to the registration and reporting of adverse events, such as regular inspections for the occurrence of pressure ulcers after admission, and regular weighting of patients to reveal malnutrition.

The aim of this article is to investigate the implementation of a patient safety programme in a hospital department to consider how the programme has affected the professional identity of nurses.

### Theory

We follow Fournier [1] and McLaughlin and Webster [2] in understanding the professional field as constituted by professional practice, and as always in motion, expandable and malleable. Implications of changes in context (for example introducing a patient safety programme) must thus be investigated in each case. Professions adapt to changes in various ways, and, as a consequence, professional boundaries are negotiated and renegotiated [22, 23]. Currie et al. [24] argued that professional identity is relational, thus a profession’s legitimacy has to be constructed actively and in relation to others. We argue that professional identity is crucial for understanding how the professions are constructed and reconstructed, and for investigating professions’ responses to changes.

Professional identity may be linked to the work and daily routines performed at a unit. Autonomy is an important part of this, and different forms of autonomy therefore play a part in professional identity. Autonomy is often defined as ‘self-governance’ and ‘self-regulation’. ‘Independence’ and ‘freedom’ are alternative definitions, although they do not share all the characteristics [25]. Taking responsibility and/or the capacity to make decisions is also important in the autonomy concept. Some authors distinguish between different forms of autonomy. Timmermans and Berg [26] differentiated for instance between what they call professional and clinical autonomy. Professional autonomy marks the parameters of clinical autonomy, while clinical autonomy may be understood as the framing of everyday work activities. A profession’s power is normally defined by clinical autonomy (idem). Everyday work activities are therefore important for the constitution of professional identity.

### Audit systems and trust

Patient safety systems are often based on approaches that are traditionally depicted as ‘audit systems’, that is, systems based on measurements and accountability, as they rely on standardised routines and guidelines for the work of health personnel. The introduction of these types of standards can also imply that trust is thought of as something that is built into standardised systems instead of being directed to professionals or experts [27, 28]. These programmes are often placed within a managerialist perspective, contrasted with professionalism [29]. Many researchers have indicated a broader movement met with resistance from health professionals, referred to by Timmermans and Berg [30] as “a push from autonomy to accountability”. This movement is international and involves pressure to delimit clinical autonomy and professional assessment, focusing mainly on the physician role [31]. While there is some knowledge about the medical profession’s relationship with audit systems, very little is known regarding the nursing profession. Some researchers [26, 29] have highlighted the adverse effects of managerialist programmes. This suggests that nurses respond to such programmes in similar ways, and researchers such as Rao et al. [32] have found that better nursing autonomy has a direct positive effect on patient safety.

New public management (NPM) is by many considered as the underlying premise of several public reforms in Norway today, so also in the health care sector. It implies an attention to accountability and cost efficiency through the use of measurements, indicators and audit systems [33]. This has met some resistance from health professionals in Norway [34]. Some researchers have argued that medical professionals have used tactics working against NPM-style reforms, integrating them only superficially and at the local level [35].

The underlying reason for the resistance from professionals against audit systems is often related to how such systems challenge professionals’ knowledge, the way in which they work and their identities [26, 34, 36].

### Tacit knowledge and invisible work

Specialised skills and mastery are important parts of professionals’ knowledge, as well as working autonomously. This has led to a formation of boundaries between themselves, other professions and the rest of the community [37]. Practice is also an important aspect for the professional practitioner [38]. Routines are often adjusted to adapt to practical requirements, which entails that they are rarely performed entirely according to procedures. Much of professional expertise is thus difficult to explicate and may be labelled as tacit. In fact, tacit knowledge, as formulated by Polyani [39], is potentially more important for professionals than explicit knowledge. Tacit knowledge involves knowledge that cannot necessarily be explicated but can be employed in practical situations. It is described as ‘know-how’ as opposed to ‘know-that’ and constitutes an important characteristic of expertise [40]. Although the work of professionals may be excellent, it may be a challenge to communicate or explicate what they have undertaken [6].

Professional practice thus tends to exclude actors outside a boundary of tacit knowledge. Other practitioners do not easily cross this boundary, which has led to a notion of untouchability and professional power [26]. It has also led to a perception in general of professionals resisting the explication of knowledge, especially to third-party actors, in the wish to maintain their knowledge esoteric [41]. Administrative indicators and measurements may be thought of as a challenge in this matter [31]. The way that knowledge development hinges on the ability to translate between tacit and explicit knowledge is also important. Nonaka and Takeuchi [42] described that knowledge creation is a process of conversion between tacit and explicit knowledge (also known as the SECI model), starting with socialisation (tacit–tacit); externalisation (tacit–explicit); combination (explicit–explicit); and internalisation (explicit–tacit).

Nursing has traditionally been characterised by invisible work, and making invisible work visible can be seen as a strategy for professionalisation and legitimacy. In our project, the introduction of the patient safety programme represents a change in context, implying new negotiations of visible/invisible work. Nurses, who are very visible as workers in a hospital, can still face challenges in constructing arenas of voice for making their work visible [43]. By changing work under the general label of ‘care’ into work that is specifically defined, legitimate and traceable across settings, they can make invisible work visible. Visibility can lead to legitimacy, but can also create the reification of work, opportunities for surveillance and an increase in paperwork [44]. Bowker, Star and Spasser [45] argued that “only work that is visible can truly be identified as valuable”. At the same time, they show that the visible representations of work in a standardised system might not reflect the process-oriented nature of nurses’ work, and such representations could imply the reassignment of ‘unskilled’ parts of nursing, resulting in less need for professional knowledge. Star and Strauss [44] argued that “nurses struggle to be visible, but simultaneously to hold areas of ambiguity and of discretion”.

Allen [17] especially drew attention to what she called ‘organising work’ – activities performed by nurses to make things ‘go round’ in health care systems and ‘glue’ them together. Such activities are largely ‘taken-for-granted’ (at least until something goes wrong), despite being an important part of the nursing role. Some have estimated that ‘organising work’ accounts for more than 70% of nursing activity [46].

### Professional values, hierarchies and status

The bio-medical knowledge tradition of doctors is renowned for explicating its knowledge and thus contributes to some of its success [47]. It is also recognised for maintaining the knowledge boundary, keeping knowledge less accessible to others (through, for instance, the use of Latin and Greek) [48]. The nursing knowledge tradition has not developed in the same way, with less attention paid to codified and standardised knowledge and more to the holistic and phenomenological side. Medicine is situated in the positivist natural science tradition, while nursing pertains to constructivist social science [49]. Traditionally, nurses have had an oral tradition regarding the transfer of skills and knowledge [50], while doctors have also relied on codified knowledge developed through extensive programmes to provide evidence [47].

The difference between the practices of nurses and doctors has also been well documented. This involves that a doctor will normally work with patient data, using talk as complementary (and secondary) input, whereas talk and conversations with patients are the primary tools of nurses [34, 51]. Nursing practice tends to use a more holistic approach to medical treatment, while doctors tend to rely on data from tests and consultations [51]. Some have described this difference via the notion that nurses treat the patient (care), while doctors treat the disease (cure) [52]. It is further important to emphasise that there is a hierarchy between these approaches, which has been named medical authority by some [53, 54] and entails medical doctors having the final word in questions of treatment. Reductionist approaches, as in the bio-medical tradition of doctors, have traditionally been given authority over holistic approaches, as in the tradition of nursing [55].

## Methods

### Study design

We followed a nursing unit for approximately four months, applying qualitative interviews as the main approach, supplemented by observations. At the time of our visit to the department, the new patient safety programme had been active for some time. This did not allow us to capture in situ observations or reflections at the time of implementation. On the other hand, it allowed us to investigate perceptions of changes and an approximation of their stabilisation for the nurses. We employed individual, in-depth interviews to study interviewee perceptions, opinions and meanings as a way to explore experiences from a holistic point of view [56]. Group interviews were employed as a supplement. According to Casey and Krueger [57], focus groups provide a “a more natural environment than that of individual interviews because participants are influencing and influenced by others - just as they are in real life”. Different meetings were observed during the four month period to gain direct insight into the concrete practices that the patient programme instigated. Combining different qualitative methods and different kinds of data has been suggested as particularly appropriate for claiming validity in qualitative research [58].

The interview guide (please see Additional file 1 Interview guide) was developed based on our interest in exploring how the national patient safety programme unfolded in a local context. The meetings observed also provided input to topics that we wished to explore further, including how the patient safety programme influenced professional identity. Informal discussions with a nurse and a manager at the beginning of the four-month period were also a source of information for developing the interview guide.

### Data collection

We conducted two group interviews with six and three nurses, four individual interviews and five hours of observational studies. Ten nurses and three persons from hospital management were interviewed. Based on purposive sampling [59], where informants are selected dependent on relevance to the project, interviewees were selected according to their availability and knowledge of the programme. The interviews lasted from one to one and a half hours, and were performed at the hospital. The interviews were semi-structured, with an emphasis on the interviewees’ perceptions. The interviews were recorded and transcribed ad verbatim. Observational studies were conducted at three different kinds of meetings involving the use of measurements and standardisations: one that was held weekly, focusing more on day-to-day tasks; another that was held monthly with greater attention to numbers; and one that involved only managers. The observational studies were conducted with an emphasis on seeing the actors in their natural setting. Field notes were taken focusing on a descriptive form, although with some elements of interpretation [60]. We therefore described events as we saw them but took some notes on our interpretation of these events. The observational studies were used as both an informative background for developing the interview guide and corroboration of elements deducted from the interviews. The interviews and observations were all conducted with two of the authors: one responsible for questioning and one responsible for taking notes. The study period was from December 2016 to April 2017, and took place at a regional hospital in Norway.

Both the interviews and the observational studies focused on the health professionals’ adoption and use of patient safety metrics and other aspects of the national patient safety programme, resistance to the programme, perception of professional identity and trust issues. Actors such as the managers, were asked to describe the development of the programme, development of indicators and what they saw as the consequences. Both observations and interviews corroborated important patterns in the data, which the authors deemed as a sign of saturation.

### Data analysis

The data was analysed following what Kvale calls ‘ad hoc meaning generation’ [56]. This entails analysing the texts in various ways, instead of following pre-decided and common routines. The analysis was conducted with all three authors first reading the transcripts, as well as the notes from the observational studies. We further employed what Kvale calls meaning condensation [56]. This involves compressing long statements into shorter statements, preserving the original essence. Categories were then developed, initially by all the authors alone and then refined by the three authors in joint sessions. This formed the basis for a tree structure and the generation of hypotheses to guide further analysis of the texts.

We built on the framework presented by Korstjens and Moser, to ensure credibility, transferability, dependability and confirmability [61]. Credibility in our findings was supported by using different data sources. It was also supported by the authors being in the field over a prolonged period of time as participants in other projects. To ensure transferability, we sought to provide thick descriptions in the presentation of the data - describing not only behaviours and experiences but also contexts. To comply with dependability and confirmability, we strove to provide transparent descriptions of the research steps (presented earlier).

Ethical approval to conduct the study was granted by the Norwegian Centre for Research Data (NSD) (project number 52699). Informed, written consent to participate in the study was obtained from all the study participants. Participants were given the opportunity to read the written transcripts, and to give feedback on our findings.

## Results

We developed four categories during the data analysis, denoting different types of changes in the nurses’ values, working practices, and status in relation to the patient safety programme: reconstructing trust; reconstructing work; reconstructing values; and reconstructing professional status. These are presented in the following.

### Reconstructing trust relationships – from trusting professional knowledge to trusting the system

The introduction of standards in the form of patient safety programmes can imply that trust is thought of as something that is built into standardised systems instead of directed towards professionals or experts [26–28]. This means that, whereas people previously trusted health care professionals to conduct good treatment of patients based on their medical discretion, trust is now transferred to standardised systems and routines. It is, to a larger extent, the standardised routine - and the documentation of this - that should guarantee a patient’s safe treatment in the hospital. These perspectives on safety work were expressed by our informants in different ways, for example by one of the nurses in a group interview:

If the documentation is not there, it [the work] has not been done. And that is what has also changed. (Group Interview 1 with six nurses).

The nurse explained that documentation was even more vital after the introduction of the patient safety programme and that it is ‘proof’ that the required work has been performed. It is not enough to conduct patient safety work or to say that it has been carried out (trust in professionals): it has to be documented (trust in systems). This is in line with the perspectives linking managerialism to NPM and the argument that NPM is associated with a rise in audit systems, which leads to ‘audit rituals’ of verification to produce government and societal confidence [35].

A similar perspective was also offered by a manager, with an emphasis on measurements:

What you cannot measure, you have no good possibility to do something about. (Hospital management).

The manager connects documentation and measurements to the ability of managers and health personnel to change work practices and perform better. He argues that to address patient safety (improve it) it is necessary to measure something. Measurements can thus be seen as trusted signs that patient safety is being addressed (or not), and as tools for improvement.

Documentation and measurements form an important part of a system built around standardised routines and guidelines. Standardisation can make medical practice more transparent (trust is implied through transparency) [26]. Nursing practice is made more transparent to managers and patients, but one of the nurses also pointed out that their practice and knowledge can be made more transparent even to co-workers:

It is not that we have not done this before, but now it is systematised, and that system also makes us cover almost all patients, and makes the nurses feel safe. You can have someone who has worked here for thirty years, and someone who has worked here for one year, and to them the system is easier to relate to in a new work practice. In addition, as a patient, you get the same nurse, if you understand what I mean. (Group Interview 1 with six nurses).

The nurse explained that the patient safety programme, in addition to making the patients safe, also makes the nurses trust that they are performing their job properly. For new nurses with less experience in particular, it is easier to relate to a transparent and standardised guideline. This also ensures, the nurse argued, that patients have ‘the same nurse’ (meaning the same treatment) independently of the nurse being less or more experienced, indicating trust in systems instead of professional knowledge. Traditionally, nurses have had an oral tradition regarding the transfer of skills and knowledge [50]. This implies that nurses’ professional knowledge has a tacit dimension [39] that is difficult for new and less experienced nurses to grasp. With the introduction of the patient safety programme, tacit knowledge can be made explicit to a larger extent, much in line with Nonaka and Takeuchi’s description of tacit knowledge being externalised into articulated knowledge in one part of their knowledge conversion model [42]. Explicit knowledge can then be made tacit again through a process of internalisation. For less experienced nurses, use of the standardised system can represent a phase in their learning process in which tacit knowledge is articulated to them, making it possible to internalise it in a later phase.

On the negative side, the increased use of standardised routines and guidelines in nurses’ work may suggest that nurses experience a reduction in their professional autonomy and the potential for individual adjustments for each patient. One of the nurses in a group interview explained safety work in a clinical setting in the following way:

For example, the pressure ulcer rounds, […] then there are two persons who are going around the entire department. We are supposed to check the patients daily for pressure ulcers, and we document that as we should. But still they have to come in and check. Then I feel it a little bit… is that trust in us? […] We cannot use our medical gaze anymore, cannot think for ourselves. Which is both positive and negative, of course. (Group Interview 2 with three nurses).

The nurse stated that their opportunities to use their professional knowledge and discretion has been limited. This can also be experienced as distrust [from the employer], as explained by a nurse:

It’s a little bit like … ok, we do not do a good enough job. I can feel a bit like that. Because I do my job, and then I expect my employer to trust that I do it. (Group Interview 2 with three nurses).

The nurse explained that the introduction of measurement and control gives her a feeling that she does not conduct her work well enough, and she argues that her employer should trust her and her work without having to rely on measurements. Professional autonomy is a vital part of the role and identity of health professionals [25], and a profession’s power is normally shown by the autonomy that its members have in everyday work [26]. The introduction of standardised systems, regulating the details of everyday work for nurses, can thus represent a threat to this autonomy/power and be in conflict with professional identity.

The introduction of the patient safety programme implies that measurement and control are given more attention and importance. Whereas the nurses were previously trusted because of their professional judgement and patient safety was an integrated part of their work, they now have to document how they work with patient safety in a predefined, systematic way. This is experienced as both positive and negative. The nurses valued the systematic work and explained how these systems can make them trust their own work and can make their (often) tacit knowledge more explicit to others, however, some also articulated a feeling of distrust from their employer and argued that they should be trusted as professionals, independently of the documented measurements.

### Reconstructing work – from invisible to visible work

Nursing work has traditionally consisted of a considerable amount of invisible work, which some researchers estimate as high as 70%: work that makes the organisation function but that is noticeable only when something goes wrong or unplanned events happen [17]. This creates challenges in the form of legitimacy; only work that is visible is valuable [44]. With the introduction of the patient safety programme, the explication of actions contributed to making more of the nurses’ work visible – thus contributing to making their work more valuable. One nurse said:

We have been doing patient safety work all the time, it is just that we have not systematised it. And we have not discussed and talked about it, as we do now. (Group Interview 1 with six nurses).

Nurses reported that documentation of patient safety work and the use of metrics and indicators allowed them to translate previously tacit nursing activities into something to which other professions could relate. One nurse recounted from a conversation with a doctor:

I was going through all the things we did as nurses, and then it was like ‘Oh my God – do you do all this!’ They didn’t know this. (Group Interview 1 with six nurses).

This illustrates one of the very important motivators for making work visible. As we pointed out earlier, a professional involves and employs a substantial amount of tacit knowledge in their practical work, which they cannot easily explain afterwards [62]. What they undertake is not necessarily transparent or understandable for others – a point that is even more valid for professions with tacit work practices and knowledge boundaries [41] and maybe even more so for the nursing profession, relying heavily on the oral form of knowledge transfer. When working together with other professions, however, this visibility of work actions contributes to making the profession’s work more valuable.

Making their work visible involved activities that the nurses normally did not perform. Measuring activities entailed time and effort, and sometimes they involved time that nurses would rather have spent with a patient. Largely, however, this was interpreted positively, because patient safety had now turned into a more palpable phenomenon. Managers would argue that, while they previously (before the programme) could be asked about the status of patient safety and not have an answer, they were now able to give clear answers based on the numbers. In other words, they felt more in control. Making patient safety work more visible contributed to the uncovering of previously unaccounted errors. For instance, there was previously a ‘concern’ among the nurses that medications were wrongly administered. This was, however, based more on a feeling or hunch or maybe even rumours. Now, with numbers, they could:

… analyse the data, categorise it, and risk analyse it … we do also see where the problems are, namely preparation and administration. We as nurses report the most, but also doctors … so we need to find appropriate actions. (Nurse).

Documented patient safety work also enabled cross-boundary support. Other professions, such as physiotherapists and doctors, were involved on the basis of the documentation of patient safety. Apparently, the numbers made it more interesting for doctors to engage in discussions and reflections at joint meetings such as the ‘pre-visit’. One nurse reported:

The doctors join in on our ‘pre-visit’ … then we discuss some of the things we work with, such as falls, infections, what we can do differently … doctors feel that this is extremely exciting, that we address things that are important for us. (Group Interview 1 with six nurses).

On the critical side, nurses also reported challenges in the reporting activity itself, when it opposed interpersonal relationships with patients (which we will turn to next). The nurses overall welcomed the programme, however, on the basis of making visible practices that were previously tacit. This involved important benefits of heightened recognition from other professions, easier cross-boundary collaboration and the systematisation of safety deviations.

### Reconstructing nursing values – from ‘care’ to ‘cure’

Nursing practices are rooted in an approach to medical treatment which can be described as holistic, while doctors are inclined to rely on data from consultations and tests [49]. This difference is sometimes described with the notion that doctors treat the disease while nurses treat the patient [52]. Some advocate that doctors adhere to ‘cure’ while nurses adhere to ‘care’ [51]. Thus, nurses tend to approach patients in a different way from doctors, relying more on dialogue and mental support, and in some instances establishing a closer relationship with them than doctors. Nursing values are thus often rooted in holistic and immeasurable care for patients, contrasted with the reductionist and quantifiable cure of doctors. The safety programme challenged these values in a way that we will describe as moving from ‘care’ to ‘cure’.

The traditional holistic approach was challenged by the quantifiable aspects of the programme, especially measurement and documentation activities. Whereas they had traditionally emphasised the caring aspect with patients, for instance holding their hands, there was now a shift towards performing more of the tasks that could be counted (and be accountable). There were concerns related to the reporting activity itself, when it opposed the interpersonal relations to patients. One nurse recounted that they were supposed to measure the weight of patients every seventh day:

And I see that we struggle with keeping up with this … […] maybe if we have a focus on what is important for the patient – food, nutrition, beverage… rather than getting the patient out of the bed, in the wheelchair and up on the scales. (Group Interview 1 with six nurses).

The nurse here was pointing out not only the struggle of the measuring itself but also that the attention should be on the patient and their well-being – not on the measuring activity. The opinion that ‘real’ nursing work should be performed in relation to the patient rather than a computer screen was shared by some nurses. One nurse said the following:

I have heard nurses say that, while I am on the computer, documenting that the patient has a risk of falling, the patient is actually falling in the room. (Group Interview 1 with six nurses).

While some nurses appreciated the new ways of working, more in line with the physicians’ ‘cure’ tradition, other nurses reported that the documentation of patient safety work challenged their professional values as a nurse. This differs from what has previously been reported as problematic with similar programmes and professionals – that they resent measurement because it is seen as a form of surveillance and control by others [29, 35]. The nurses in our project seemingly put more weight on the measuring activity as a challenge to professional values. As such, it can be argued that the resentment was more a threat to their clinical autonomy (everyday practice [26]) than to their professional autonomy (markers of professional boundary [26]). Their clinical autonomy was only challenged if they could not see the measuring activity as meaningful or in other words something that they themselves judged to be important.

The administration and coordination side of nursing has necessarily always involved some form of documentation [17], however, this was now taken to a different level. The nurses themselves resented the measuring activity itself – not the measures themselves – but on the other hand needed to develop, adjust and adapt to these new demands.

Nurses were approaching the physicians’ way of using measurements in their own work, wanting evidence about whether and how their activities were working. Although their aims and means were different, nurses were influenced by the programme into working towards a more measurable way of curing the disease. The more difficult-to-measure caring activities of, for instance, holding hands and developing meaningful conversation, could in some sense be downplayed. The visualisation of patient safety work through numbers is in accordance with a ‘cure’ discourse, also contributing to a heightened professional status which we will turn to next.

### Reconstructing professional status – from lower to higher (perceived) status

The nursing profession has in many instances been contrasted with that of physicians and the ‘care’ and ‘cure’ dichotomy [51]. Nurses’ practices have been described as holistic, while doctors’ practices are referred to as more specific, and as data driven by tests and consultations [49]. The status differences related to the responsibility and medical authority of physicians have been discussed extensively [53, 54].

As illustrated above, the reconstructions that originated from the patient safety programme were perceived as both positive and negative by the nurses. Several of the informants explained that the programme overall had heightened the nurses’ professional status. Some of the ways in which trust, values and work were reconstructed seem to be accompanied by the strengthening of the nurses’ professional self-esteem and the perception of their professional status. In general, the informants expressed considerable pride in what they had accomplished in relation to the patient safety programme:

I think people are proud of being as good in patient safety as we are. We have a good score on the patient safety culture surveys. And discussing incidents and errors is encouraged. (Group Interview 1 with six nurses).

The nurses related their accomplishments to an improved safety culture and open discussions about safety issues and errors. The improved professional pride can partly be regarded as a consequence of the increased visibility of their patient safety efforts. The nurses explained that:

I think it has put us on the map on a national level. It has a great significance for the employees here, that we can congratulate ourselves with something. (Group Interview 1 with six nurses).

The nurses’ newly won ability to refer to numbers in describing patient safety in a clinical setting was important – it allowed them to gain attention at the national level. Ritualistic defences towards being audited reported in other contexts [35] seem to be of varying relevance in our case, possibly because of the positive effects that have been observed:

It has become a huge professional boost, because often it is difficult to define what our work tasks are. (Nurse).

The increased visibility of their work tasks was thus considered to have a motivational effect for the nurses as well. Bowker et al. claimed that only visible work can be identified as valuable, and at least some parts of the nurses’ work have become more transparent [45]. The measures of patient safety seem to have served as a visualisation of some of their tacit activities, not only to themselves but maybe more importantly to both patients and third-party actors. More precisely, the patient safety programme makes it possible to consider the quality of the selected aspects of ‘care’, much in the same way as ‘cure’ is considered. The methods for measurement are comparable and recognisable across the professions. This might give the nurses access to medical authority [53, 54], increasing their professional status.

## Discussion

**Fig. 1:**
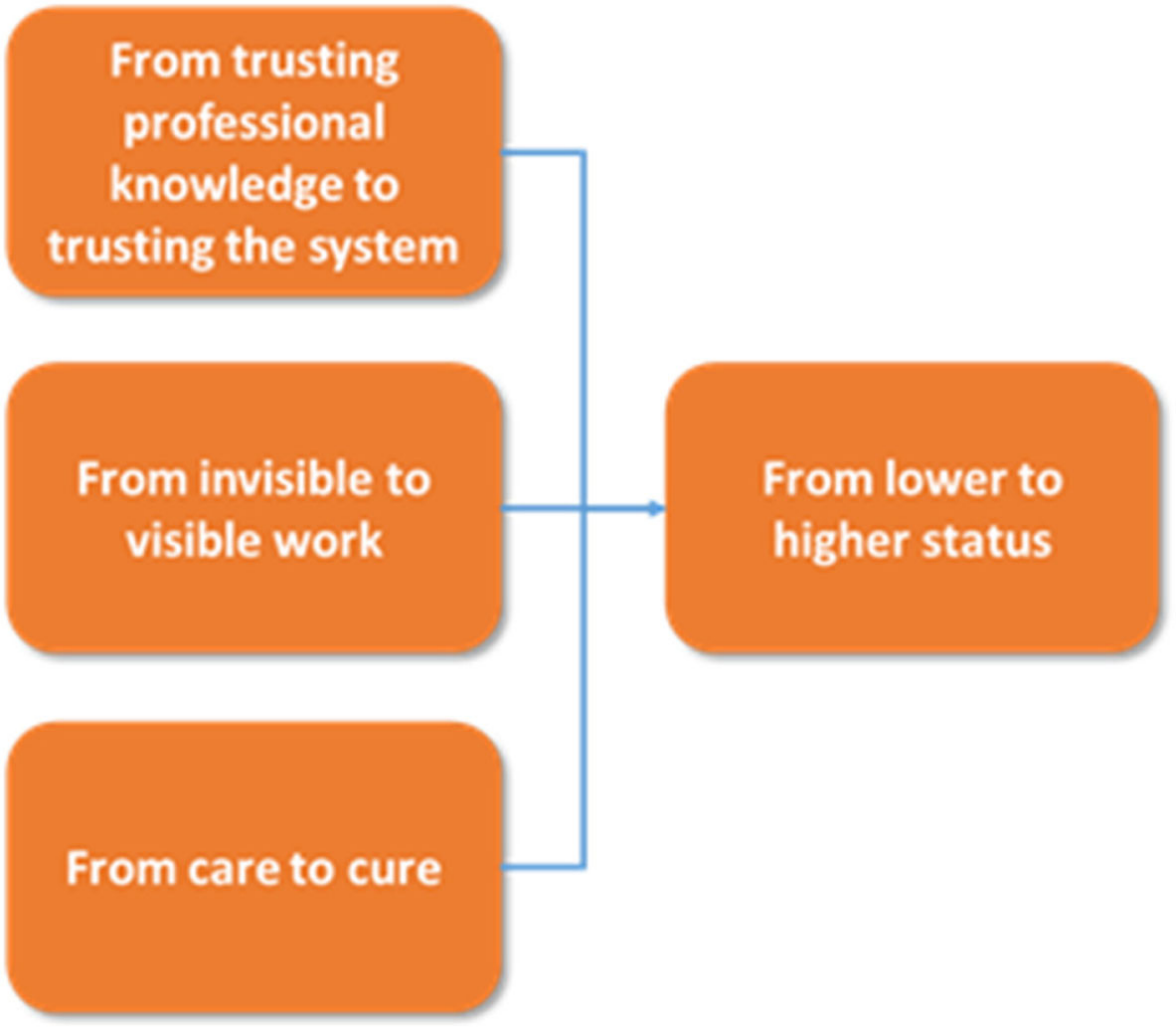
Analytical categories in the data analysis

The results illustrate that the patient safety programme involved a shift in patient safety-related decisions from being based on professional judgement to being more system-based. Further, some of the patient safety work that previously had been invisible and tacit became more visible. The patient safety programme involved activities that were more in accordance with the ‘cure’ discourse than the traditional ‘care’ work within nursing. As a result, this implied a heightened perceived professional status among the nurses. The safety programme was - contrary to the ‘normal’ resistance against audit systems - well received because of the perceived raised professional status among the nurses. The categories developed in our study and their internal relationship are illustrated in Fig. 1. The figure shows how the three first categories enable and contribute to the final category of increased status:

We argue that, through all the work involved in implementing the procedures from the safety programme, trust, work, values and status, and even the profession itself, is being reconstructed. Professional knowledge and identity are being challenged and changed; what counts as good, professional nursing of high quality is being reconstructed. There is a shift from a tradition based on broadly defined ‘care’ to a tradition in which professional nursing to a larger extent also focuses on precisely defined, visible and measurable results of the work performed.

The rise in the perceived professional status because of trust in systematised and visible work, more in line with a ‘cure’ discourse, is accompanied by some dilemmas. The nurses value their work becoming more systematised and visible, but they also expressed a concern that this might come at the expense of close contact with the physical patient and the holistic ‘care’ work, traditionally perceived as being crucial to the professional nursing identity. This holistic approach has played an important role as a counterbalance to the ‘cure’ tradition and the technology-driven medical profession [63], and one might question how losing/changing the ‘care’ perspective might affect the health care system and/or the patients.

The limitations of this study include its relatively small number of informants and limited study period, and the study could have benefited from, for example, a more extensive period with observations of actual safety practices. However, even with these limitations, we have gained important insights into nurses’ new perceptions of safety work, and moving forward, we suggest some possible future directions for the nursing profession based on our findings. Will the introduction of ever-more-specific, visible representations of work lead to the development of deskilling and the deprofessionalisation of nurses, in line with Bowker, Star and Spasser’s [45] description of such representations, possibly implying the reassignment of the ‘unskilled’ parts of nursing (less need for professional knowledge)? Following McGivern et al.’s study of how manager-professional ‘hybrids’ work to maintain and hybridise their professional identity in a managerialist context [64], do we see the beginning of nursing as a ‘hybrid’ audit profession? Alternatively, could the increased use of audit systems in nursing/the health care system advocate nurses reclaiming their traditional professional knowledge and their identity as care workers?

Following Fournier [1] and Mclaughlin and Webster [2] in understanding the professional field as being always in motion, malleable and expendable, we argue that the nursing profession is being constructed and reconstructed in relation to, influenced by, and influencing larger and more comprehensive changes within the health care system. By investigating patient safety work within the nursing profession, we can also make a contribution to understanding dilemmas concerning measurement and control, as well as professional knowledge and identity, within safety work in the health care system more in general.

## Conclusion

In a period when patient safety, often understood as audit systems, is firmly on the agenda in health care systems in many countries, it is vital to understand more about how professionals receive, understand and work with these systems and how these changes might affect the very core of their professions. This article has illustrated how a patient safety programme has affected the professional identity of nurses. The implementation has involved reconstructions of trust, work, and values, accompanied by a strengthening of the nurses’ professional status.

One important issue for further research is to examine the potential significance of such changes for the patient- nurse relationship. For patients, good quality care and a holistic approach will still be of vital importance. As the results illustrate, patient safety programmes involve a push towards accountability, and might have unintended consequences for the caring of patients. A patient perspective on this issue would be valuable and add to the understanding of the consequences of audit systems in health care.

## Additional file

Additional file 1: Interview Guide. (DOCX 22 kb)

## Data Availability

The data from the qualitative interviews is not made publicly available in order to fully protect the informants’ anonymity. Interview guides will be made available on request to the corresponding author.

## Abbreviations

NPM: New public management
NSD: Norwegian Centre for Research Data – a national resource centre that among other things assists researchers with issues of methodology, privacy and research ethics.
WHO: World Health Organization
