# Supplementary material for: Advancing the status of nursing: reconstructing professional nursing identity through patient safety work
Source: BMC Health Serv Res. 2019 Jun 24;19:418. doi: 10.1186/s12913-019-4222-y (PMC6591911; doi:10.1186/s12913-019-4222-y)
Supplement: Supplementary file 1 — Interview Guide. (DOCX 22 kb) [file 12913_2019_4222_MOESM1_ESM.docx]

**Interview Guide**

**Introduction**

Management and management's importance for patient safety is recognized as important; however, more research is required to address this issue in the field. Specifically, more information about how managers use security measurements for improvement, and further how non-measurable security factors are safeguarded through management is needed. In this project, we will address the role of management at different levels (strategic, clinic and department), and how nurses´ professional identity is influenced by the employment of patient safety indicators. These will be addressed in terms of the clinic's own measurements and measures in addition to the use of national indicators. The project will, where appropriate, be linked to the areas of action in the Patient Safety Program, such as pressure ulcers, SVK and postoperative infections.

**General issues about patient safety**

• Is patient safety being discussed at the department level? Perhaps in what contexts?

• What types of patient injuries are there at the department level?

• Is this something that happens often? How has the development been?

**Indicators / measurement of patient safety**

• How do you know the degree of patient safety at the department level?

• Are there any types of patient safety goals or indicators at the department level?

• What is the history of these goals / indicators? Origin, who introduced etc.

**Use and benefit of indicators**

• Do you use these goals or indicators in any way in improvement work? Possibly how?

• How do you generally assess the benefits of these indicators? Strengths / weaknesses

• Could it cause you to lose focus on other things that may be important?

• Are there other side effects / negative effects of the measurements / indicators?

**Management and trust**

• Are there other types of use for these indicators other than improvement? Surveillance?

• Can the monitoring of patient safety affect the relationship with management?

• Does it influence the working environment and camaraderie within the department?

• Does it influence communication between management and employees?

**Management and control / measurement**

• How does it seem to be measured?

• How do you motivate / motivate each other / employees to achieve goals?

• Do you experience any contradictions or dilemmas caused by security indicators?

**Areas for improvement**

• Are there any other measurement methods for patient safety that could have been used?

• Are there any improvement areas you see regarding the dissemination of results or the use of improvement work?

**Patient visits**

• Have you been in agreement with the system of patient safety visits?

• How did you experience this scheme?

• What effects do you think this scheme may have?

**General issues**

- How do you measure / get an overview of patient safety in the department? How is this used by managers at different levels and other employees?
- How is the goodness of the measurement methods for patient safety assessed at different levels?
- How are the goals of daily quality work interpreted and applied?
- What features and effects can patient safety goals have in addition to quality improvement?
- Which areas for improvement can be identified with regard to measurement and application of patient safety goals?
- What effect do patient safety visits have?
